# Supplementary material for: Antiviral drugs prolong survival in murine recessive dystrophic epidermolysis bullosa
Source: EMBO Mol Med. 2024 Mar 10;16(4):870–84. doi: 10.1038/s44321-024-00048-8 (PMC11018630; doi:10.1038/s44321-024-00048-8)
Supplement: Supplementary file 1 — Appendix [file 44321_2024_48_MOESM1_ESM.pdf]

Appendix Table of Content

Appendix Figure S1. Visual of matrix detachment……………………………………………........……2

Appendix Figure S2. Antivirals screen identifies new targets for fibrosis prevention in RDEB………...….3

Appendix Figure S3. Concentration curve for antiviral hits……………………………………….…4

Appendix Figure S4. Antivirals do not affect non-EB matrix detachment or proliferation…………………5

Appendix Figure S5. No impact on collagen processing enzymes with treatment……………………….....6

Appendix Table S1. 43 compounds that delayed detachment…………………………………...………..7

Appendix Table S2. 32 antivirals that delayed detachment……………………………………………….....8

Appendix Table S3. Patient cells used in this study…………………………………………………………9

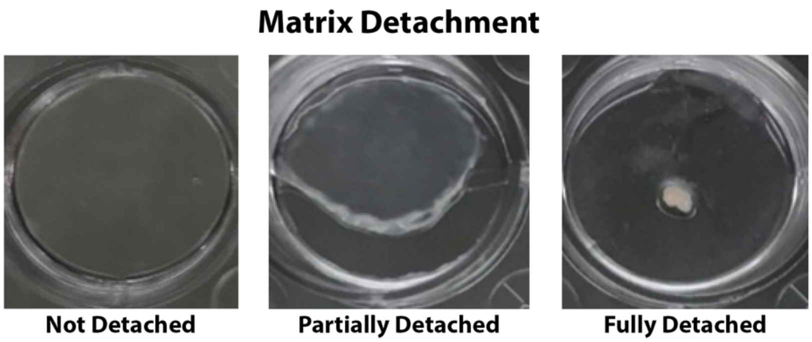

Appendix Figure S1. Visual of matrix detachment. Matrices in the process of detachment, from formed but attached, to partially detached from the well, and full detachment from the well.

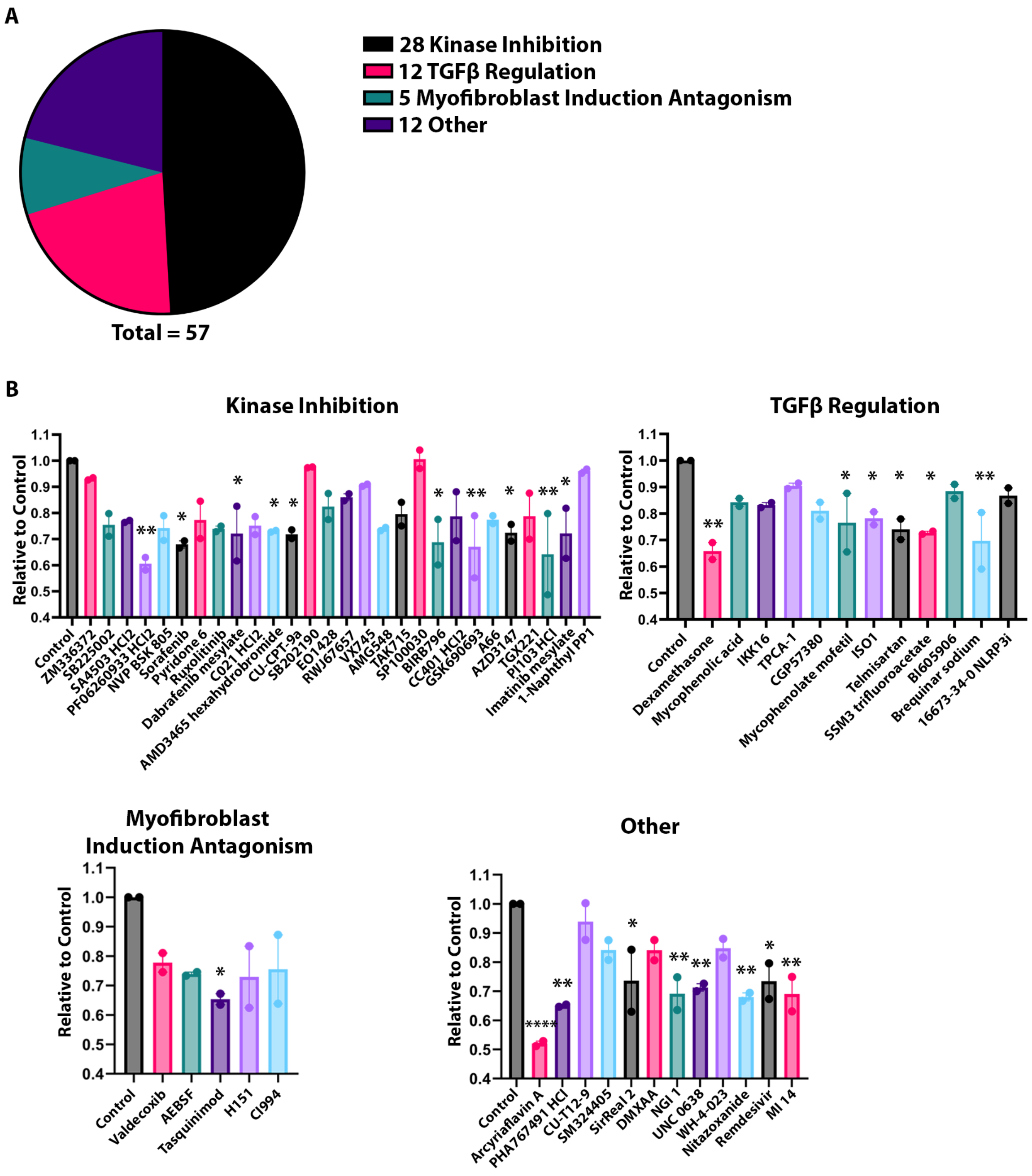

Appendix Figure S2. Antivirals screen identifies new targets for fibrosis prevention in RDEB. (A) Pie chart visual showing drug class hit-targets for the 57 of the 240 antiviral compounds that decreased time to detachment in two RDEB populations matrix assays compared to DMSO vehicle control. **(B)** Graphs showing mean ± SEM of viability for all 57 compound hits that delayed detachment relative to DMSO control in both RDEB patient populations (n=2 biological replicates) stratified by drug class shown in (A). For all panels, one-way ANOVA performed with Dunnett correction for significance: *p<0.05; **p<0.01; ****p<0.0001.

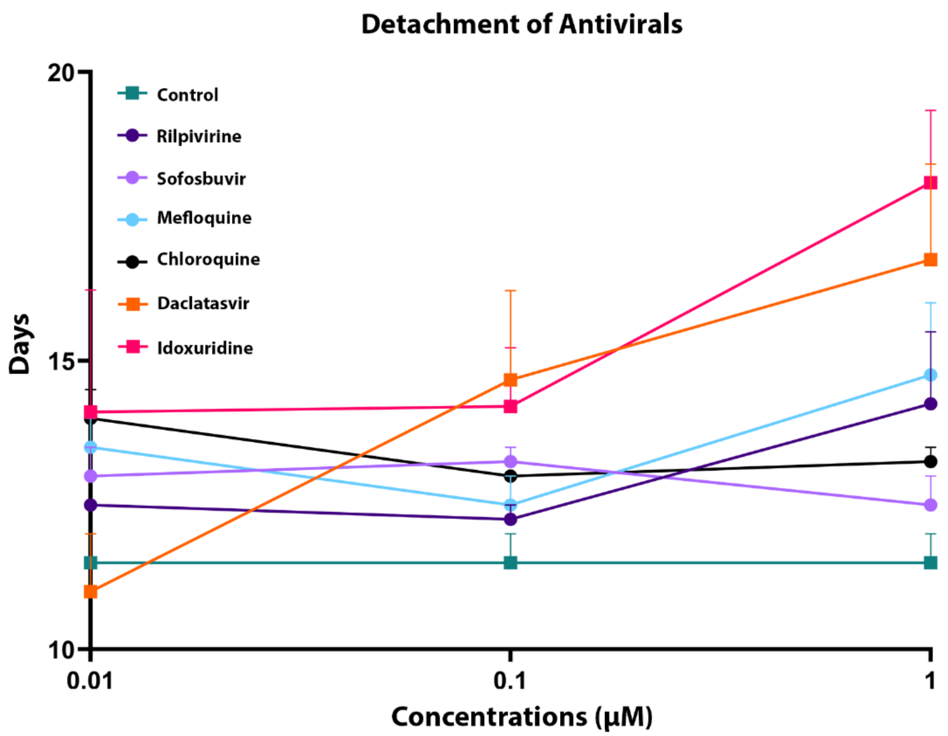

Appendix Figure S3. Concentration curve for antiviral hits. Time-concentration curve of RDEB matrix with vehicle control, rilpivirine, sofosbuvir, mefloquine, chloroquine, daclatasvir, and idoxuridine (n=2 biological replicates, mean± SEM). Ordinary one-way ANOVA with Dunnett test performed for significance, ns: $p \geq 0.05$.

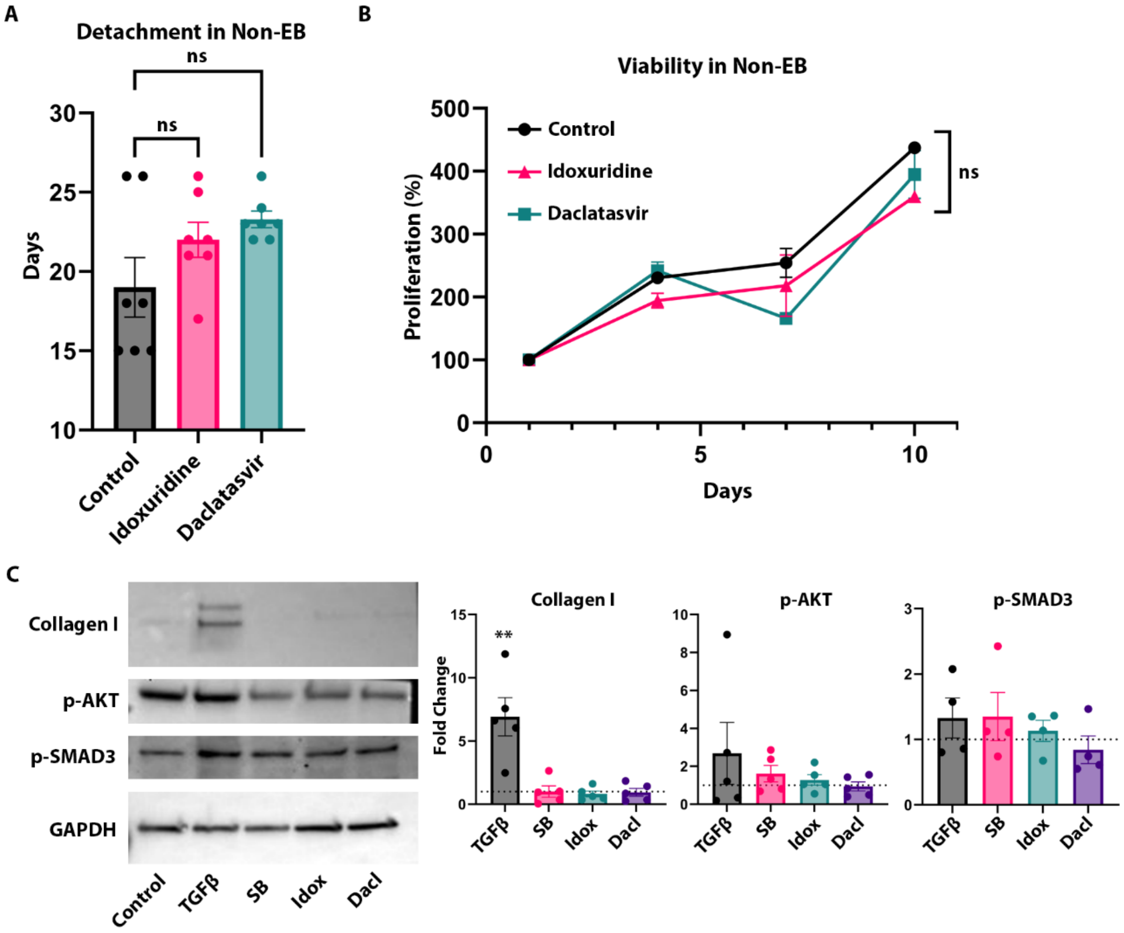

Appendix Figure S4. Antivirals do not affect non-EB matrix detachment or proliferation. (A) Graph showing mean ± SEM of time to matrix detachment in non-EB fibroblast populations comparing vehicle control (n=7, 2 biological replicates in 3-4 technical replicates) to idoxuridine (n=7, 2 biological replicates in 3-4 technical replicates) and daclatasvir (n=7, 2 biological replicates in 3-4 technical replicates) treatment. Kruskal-Wallis test with Dunn's correction performed for significance. **(B)** Line graph showing mean ± SEM of non-EB cell proliferation as a percentage compared to Day 1 with vehicle control (n=4, 2 biological replicates in duplicate), idoxuridine (n=4, 2 biological replicates in duplicate), and daclatasvir (n=4, 2 biological replicates in duplicate). Ordinary one-way ANOVA with Dunnett test performed. For both panels, ns: $p \geq 0.05$. (**C**) Western blots of collagen I, p-AKT, p-SMAD3, and GAPDH in non-EB fibroblasts with exogenous TGFβ, SB431542, idoxuridine, and daclatasvir treatment (left) and quantification of blots presented as graphs showing mean ± SEM of collagen I (n=5 biological replicates), p-AKT (n=5 biological replicates), and p-SMAD3 (n=4 biological replicates) relative to GAPDH and quantified as fold change over DMSO vehicle control. RM one-way ANOVA with Friedman test for p-AKT, p-SMAD3, and collagen I; **$p<0.01$, $p>0.05$ is not significant (ns).

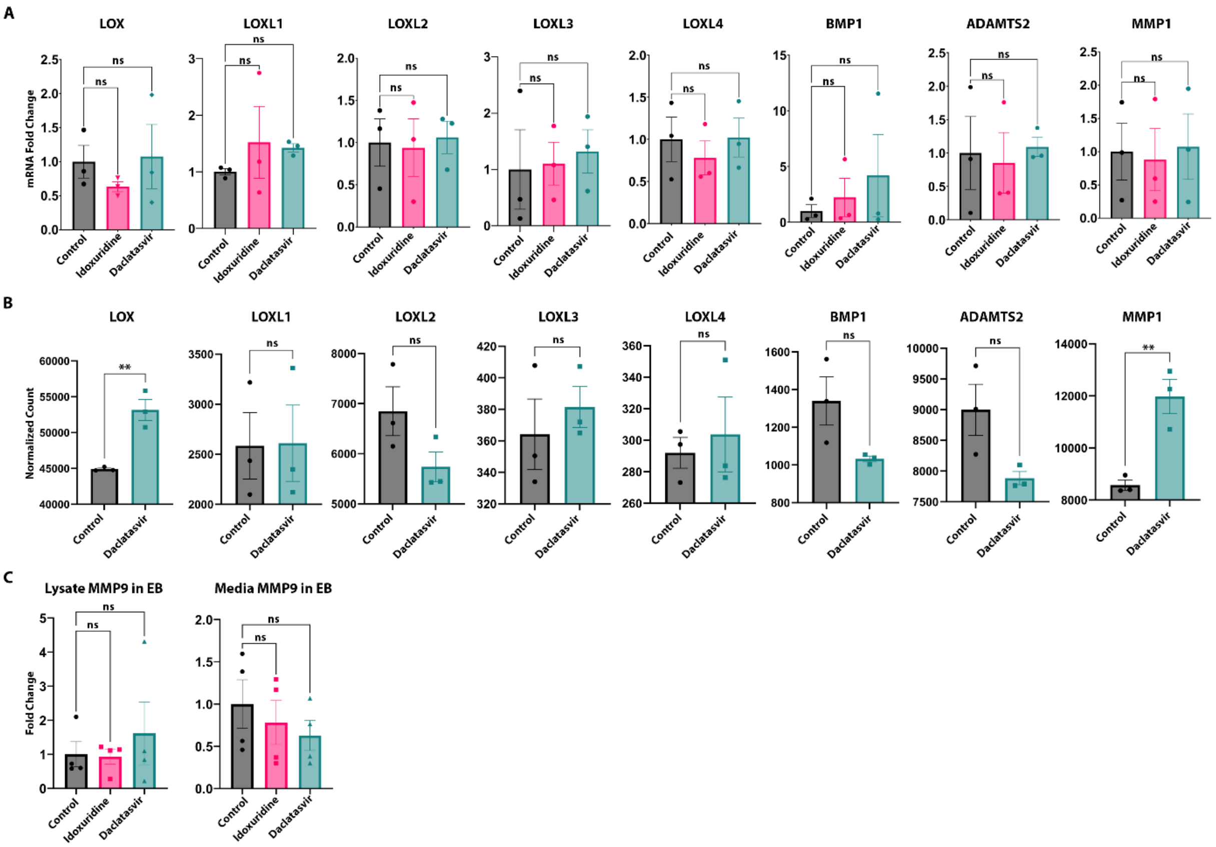

Appendix Figure S5. No impact on collagen processing enzymes with treatment. (A) Graphs showing mean± SEM of mRNA quantification of LOX, LOXL1-4, BMP1, ADAMTS2, and MMP1 for vehicle control, idoxuridine, and daclatasvir-treated EB fibroblasts (n=3 biological replicates) relative to untreated. One-way ANOVA performed with Dunnett correction for significance, ns: p≥ 0.05. (B) Graphs showing mean± SEM of normalized counts DESeq2 from RNA sequencing of LOX, LOXL1-4, BMP1, ADAMTS2, and MMP1 for vehicle control and daclatasvir-treated EB fibroblasts (1 biological replicate in triplicate). Unpaired t-test performed for significance, **p<0.01; ns: p≥ 0.05. (**C**) Graphs showing mean± SEM of ELISA protein levels of MMP9 in lysate and media for vehicle control, idoxuridine, and daclatasvir-treated EB fibroblasts (n=4 biological replicates) relative to untreated. One-way ANOVA performed with Dunnett correction for significance, p>0.05 is not significant (ns)..

Adapalene	Halcinonide
Alprostadil	Idelalisib (CAL-101, GS-1101)
Amoxapine	Idoxuridine
Aprepitant	Lapatinib
Beclomethasone dipropionate	Lapatinib (GW-572016) Ditosylate
Betamethasone	Manidipine
Betamethasone Dipropionate	Mefloquine HCl
Betamethasone Valerate	Pimobendan
Budesonide	Promethazine HCl
Chlormadinone acetate	Resveratrol
Chloroquine Phosphate	Rilpivirine
Cyclosporine	Ripasudil (K-115) hydrochloride dihydrate
Daclatasvir (BMS-790052)	Ruxolitinib (INCB018424)
Deferasirox	Sofosbuvir (PSI-7977, GS-7977)
Desonide	Solifenacin succinate
Dexamethasone (DHAP)	Thioridazine HCl
Dexamethasone Acetate	Tioxolone
Dextromethorphan hydrobromide hydrate	Tolvaptan
Eltrombopag Olamine	Triamcinolone Acetonide
Fluocinolone Acetonide	Vitamin D3
Fluticasone propionate	Vorapaxar
Fluvoxamine maleate	

Appendix Table S1. 43 compounds that delayed detachment.

ZM 336372	TGX 221
SB 225002	1-Naphthyl PP1
SA 4503 dihydrochloride	Mycophenolic acid
NVP BSK 805	IKK 16
Pyridone 6	TPCA-1
Ruxolitinib	CGP 57380
C 021 dihydrochloride	BI 605906
SB 202190	16673-34-0 NLRP3i
EO 1428	Valdecoxib
RWJ 67657	AEBSF
VX 745	H 151
AMG 548	CI 994
TAK 715	CU-T12-9
SP 100030	SM 324405
CC 401 dihydrochloride	DMXAA
A66	WH-4-023

Appendix Table S2. 32 antivirals that delayed detachment.

Patient	Cell Name	COL7A1 Mutation	Figure Reference
Breast reduction	BR23	Wild type	1E,4E
Breast reduction	BR30	Wild type	1A, 1D
Breast reduction	BR31	Wild type	1E, 1F, 1I, 4E, AF4B
Breast reduction	BR44	Wild type	1A, 1D
Breast reduction	BR47	Wild type	4E
Breast reduction	BR49	Wild type	1A, 1D
Breast reduction	BR51	Wild type	1A, 1D
Breast reduction	BR52	Wild type	1A, 1D
Breast reduction	BR59	Wild type	4E
Breast reduction	BR61	Wild type	1F, AF4C
Breast reduction	BR62	Wild type	1A, 1D
Breast reduction	BR71	Wild type	1E, 1F, 1I, 4E, AF4B, AF4C
Breast reduction	BR73	Wild type	1A, 1F, 1I
Breast reduction	BR76	Wild type	AF4A
Breast reduction	BR81	Wild type	1F, AF4C
Breast reduction	BR82	Wild type	1A, 1D
Breast reduction	BR84	Wild type	AF4A
Breast reduction	BR87	Wild type	AF4C
Breast reduction	BR92	Wild type	1F,4E, AF4C
Severe RDEB	EB70	c.425 A>G, p.K142R / c.425 A>G, p.K142R	1B, 1C
Severe RDEB	EB71	IVS34-1G>A; (+/-) 3840delC	1A, 1B, 1C, 1F, 1I, 3A, 3C-K, 4A, 4C, 4D, AF5A, AF5C
Intermediate RDEB	EB73	c.2471dupG / c.2471dupG	1F, 1I, 4C, 4D, AF5A, AF5B
Severe RDEB	EB75	Sequencing in progress	1B, 1C, 1F, 1G, 4F
Intermediate RDEB	EB80	c.2471dupG / c.2471dupG	1A, 1B, 1C, 1E, 1F, 1G, 4B, 4F
Severe RDEB	EB83	c.C4373T:p.P1458L / c.5772þ1delG	1A, 4A
Intermediate RDEB	EB85	c.2044C>T:p.R682X / c.6101G>C: p.G2034A	1A, 1B, 1C, 1E, 1F, 1G, 4B, 4F
Severe RDEB	EB99	c.6527dupC and c.5532+1G>T	1H
Severe RDEB	EB101	c.2005C>T/c.2005C>T	1H
Severe RDEB	EB102	c.8440C>T /c.8440C>T	1H
Severe RDEB	EB103	Sequencing in progress	1E, 1H, 4B, 4E
Severe RDEB	EB104	Sequencing in progress	1A, 1H, 4A, 4D
Severe RDEB	EB118	c.1732C>T:p.R578X / c.7474C>T: p. R2492X	1E, 4B, 4E

Intermediate RDEB	EB119	c.5565_5568þ8delinsA / c.6527insC:p. G2177WfsX113	1A, 1E, 1F, 2A, 2B, 2C, 2D, 3B, 3C-K, 4A, 4B, 4C, 4D, 4E, AF2, AF5A, AF5C
Severe RDEB	EB121	c.1732C>T: p.R578X / c.7786delG: p. G2596VfsX33	1A, 2E, 2F, 4A, AF2, AF3, AF5C
Severe RDEB	EB123	c.6527insC: p.G2177WfsX113 / c.6527insC: p.G2177WfsX113	1E, 2E, 2F, 4E
Severe RDEB	EB126	Sequencing in progress	1F,4C, 1I
Severe RDEB	EB135	Sequencing in progress	AF5C

Appendix Table S3. Patient cells used in this study.
