# Supplementary figures and images for: Antiviral drugs prolong survival in murine recessive dystrophic epidermolysis bullosa
Source: EMBO Mol Med. 2024 Mar 10;16(4):870–84. doi: 10.1038/s44321-024-00048-8 (PMC11018630; doi:10.1038/s44321-024-00048-8)

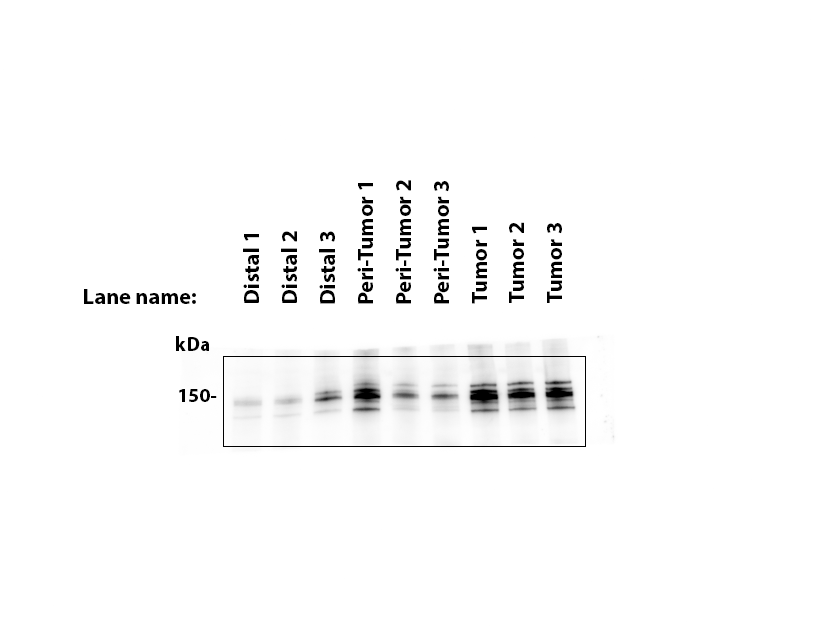

Supplement: Supplementary file 3 — Source Data Fig. 2 [file 44321_2024_48_MOESM3_ESM.zip › Figure 2/2D/Collagen I blot.tif]

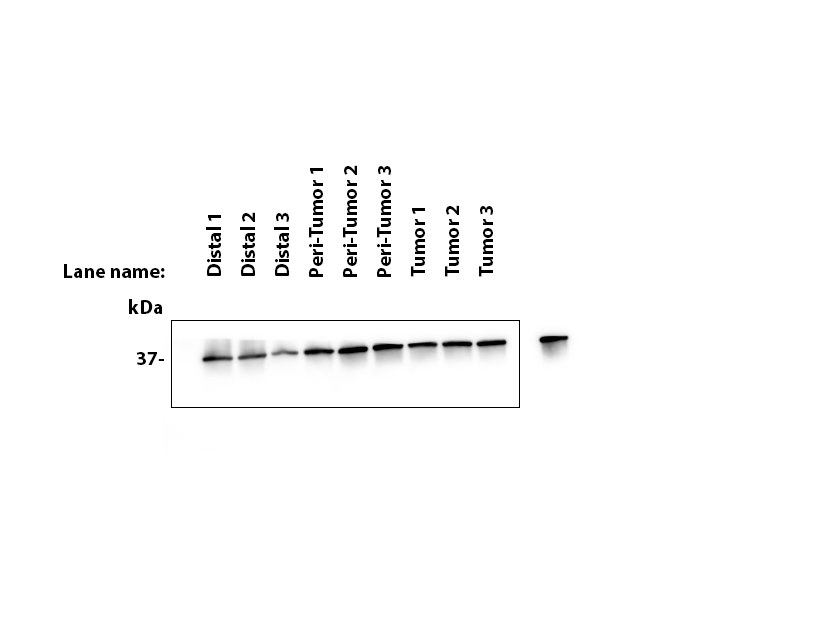

Supplement: Supplementary file 3 — Source Data Fig. 2 [file 44321_2024_48_MOESM3_ESM.zip › Figure 2/2D/GAPDH blot.tif]

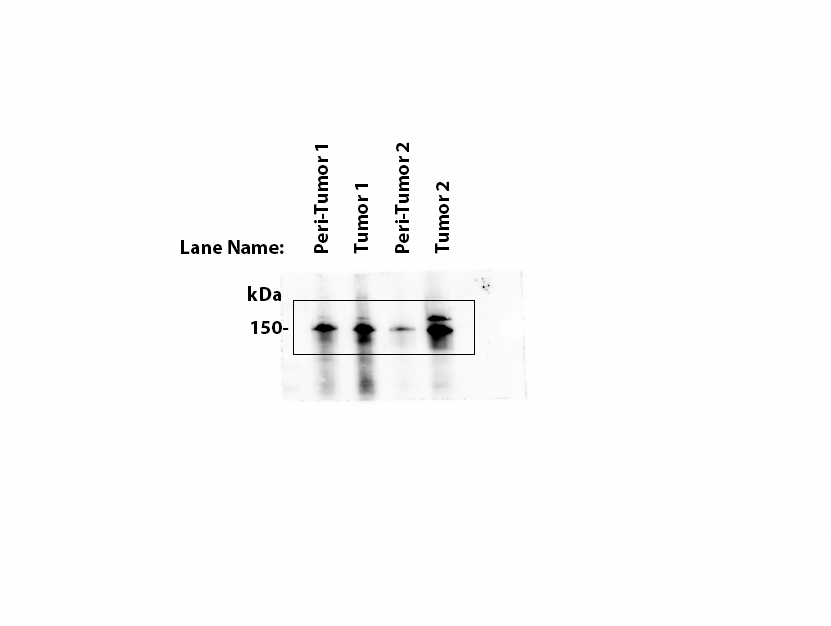

Supplement: Supplementary file 3 — Source Data Fig. 2 [file 44321_2024_48_MOESM3_ESM.zip › Figure 2/2E/Collagen I Blot.tif]

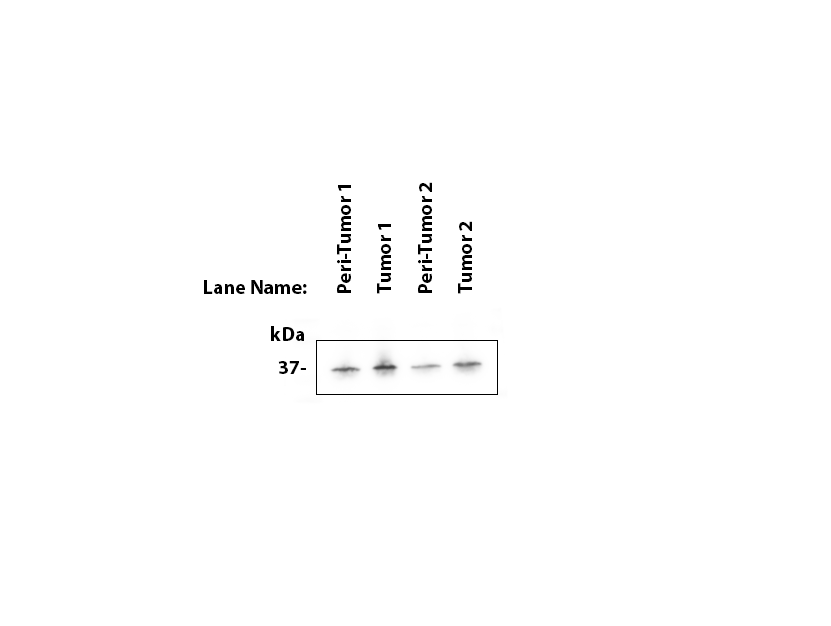

Supplement: Supplementary file 3 — Source Data Fig. 2 [file 44321_2024_48_MOESM3_ESM.zip › Figure 2/2E/GAPDH Blot.tif]

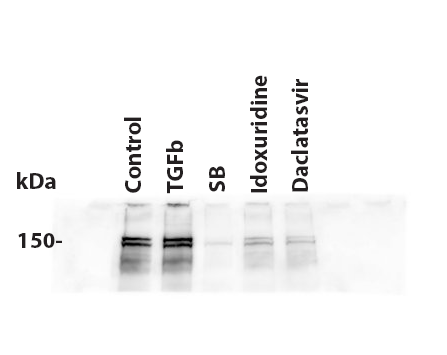

Supplement: Supplementary file 5 — Source Data Fig. 4 [file 44321_2024_48_MOESM5_ESM.zip › Figure 4/4C/Collagen I Western.tif]

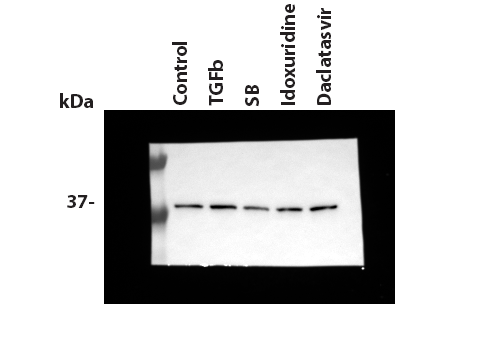

Supplement: Supplementary file 5 — Source Data Fig. 4 [file 44321_2024_48_MOESM5_ESM.zip › Figure 4/4C/GAPDH Western.tif]

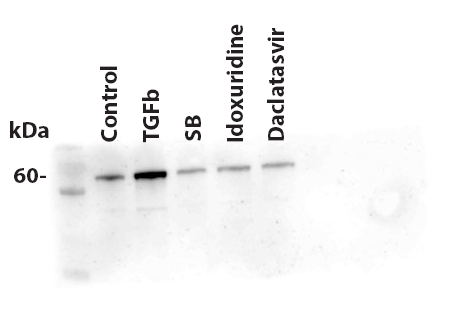

Supplement: Supplementary file 5 — Source Data Fig. 4 [file 44321_2024_48_MOESM5_ESM.zip › Figure 4/4C/pAKT Western.tif]

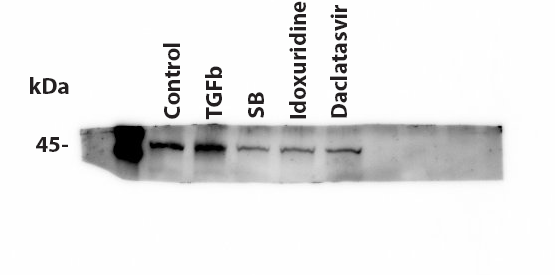

Supplement: Supplementary file 5 — Source Data Fig. 4 [file 44321_2024_48_MOESM5_ESM.zip › Figure 4/4C/pSMAD3 Western.tif]

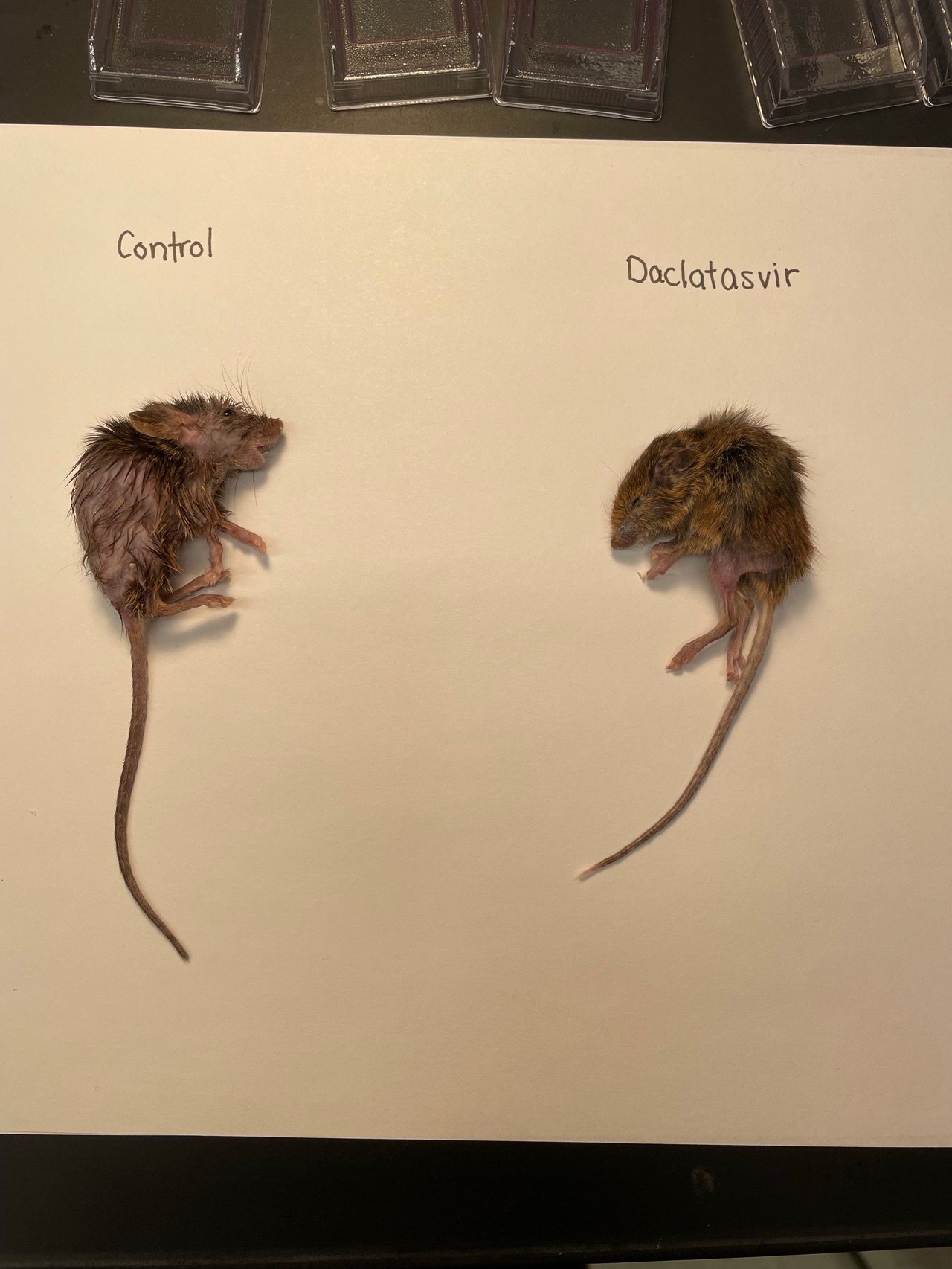

Supplement: Supplementary file 6 — Source Data Fig. 5 [file 44321_2024_48_MOESM6_ESM.zip › Figure 5/5D/hair loss.jpg]

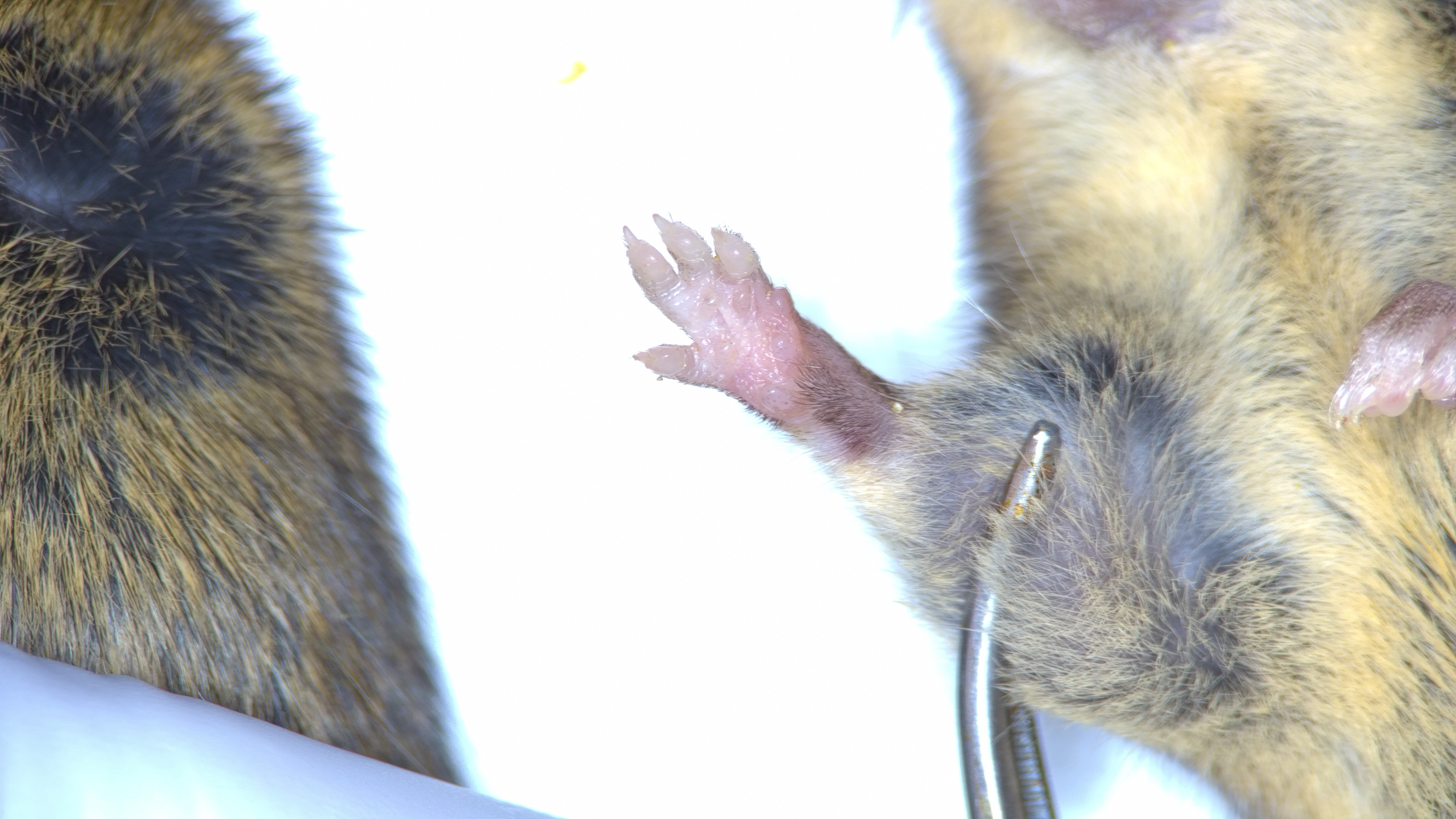

Supplement: Supplementary file 6 — Source Data Fig. 5 [file 44321_2024_48_MOESM6_ESM.zip › Figure 5/5E/Daclatasvir treated.png]

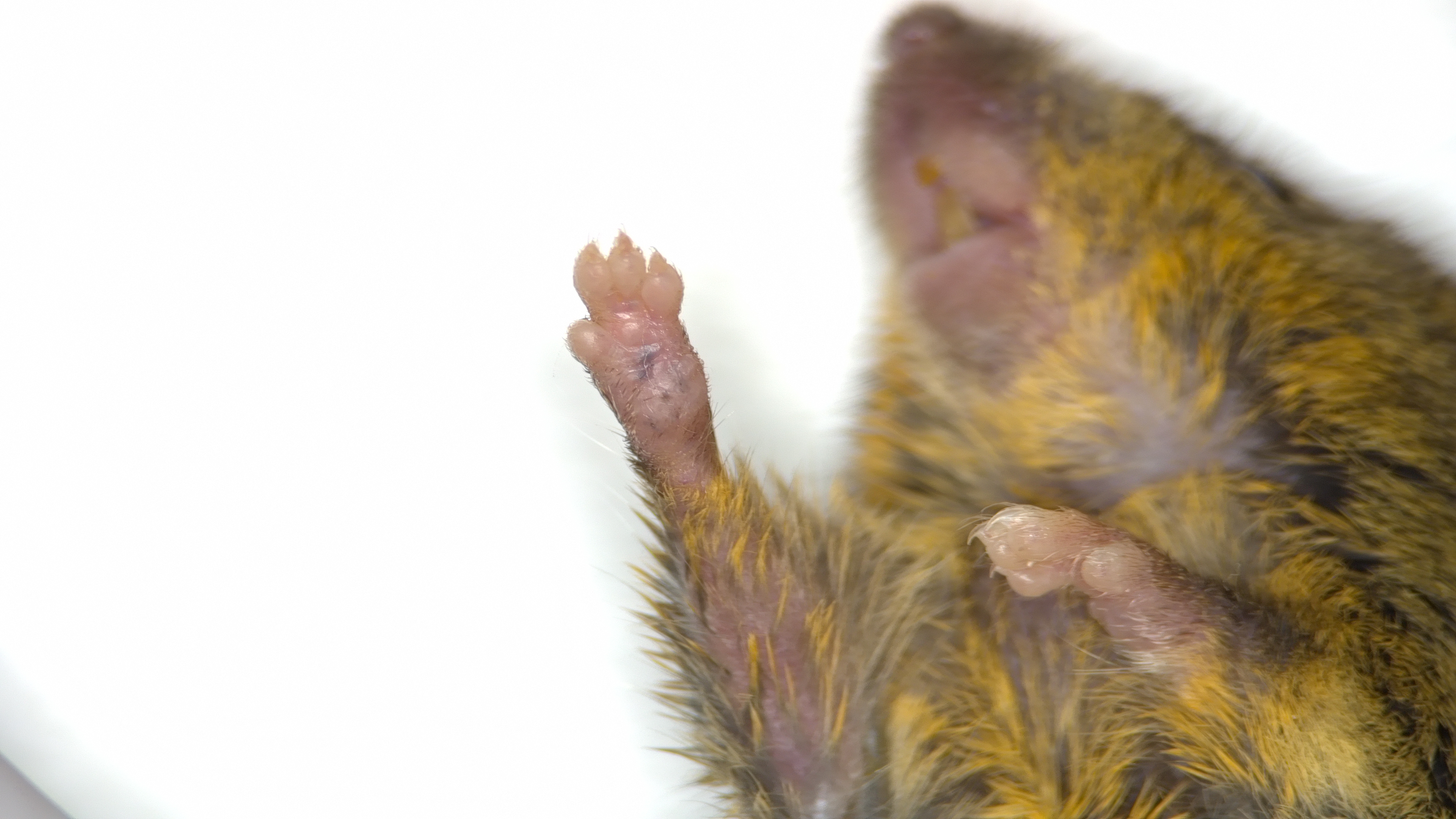

Supplement: Supplementary file 6 — Source Data Fig. 5 [file 44321_2024_48_MOESM6_ESM.zip › Figure 5/5E/untreated.png]

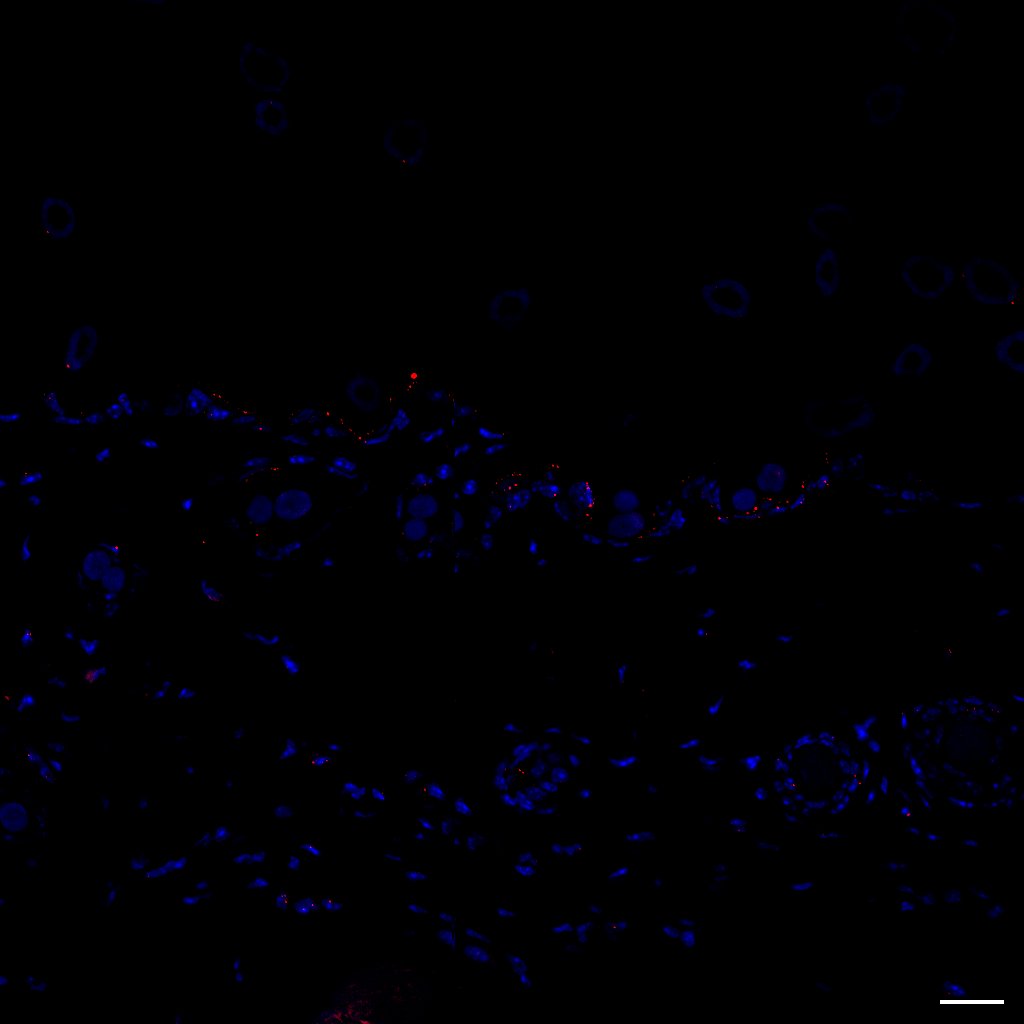

Supplement: Supplementary file 6 — Source Data Fig. 5 [file 44321_2024_48_MOESM6_ESM.zip › Figure 5/5F/EB + daclatasvir skin (r)pSMAD3 composite.jpg]

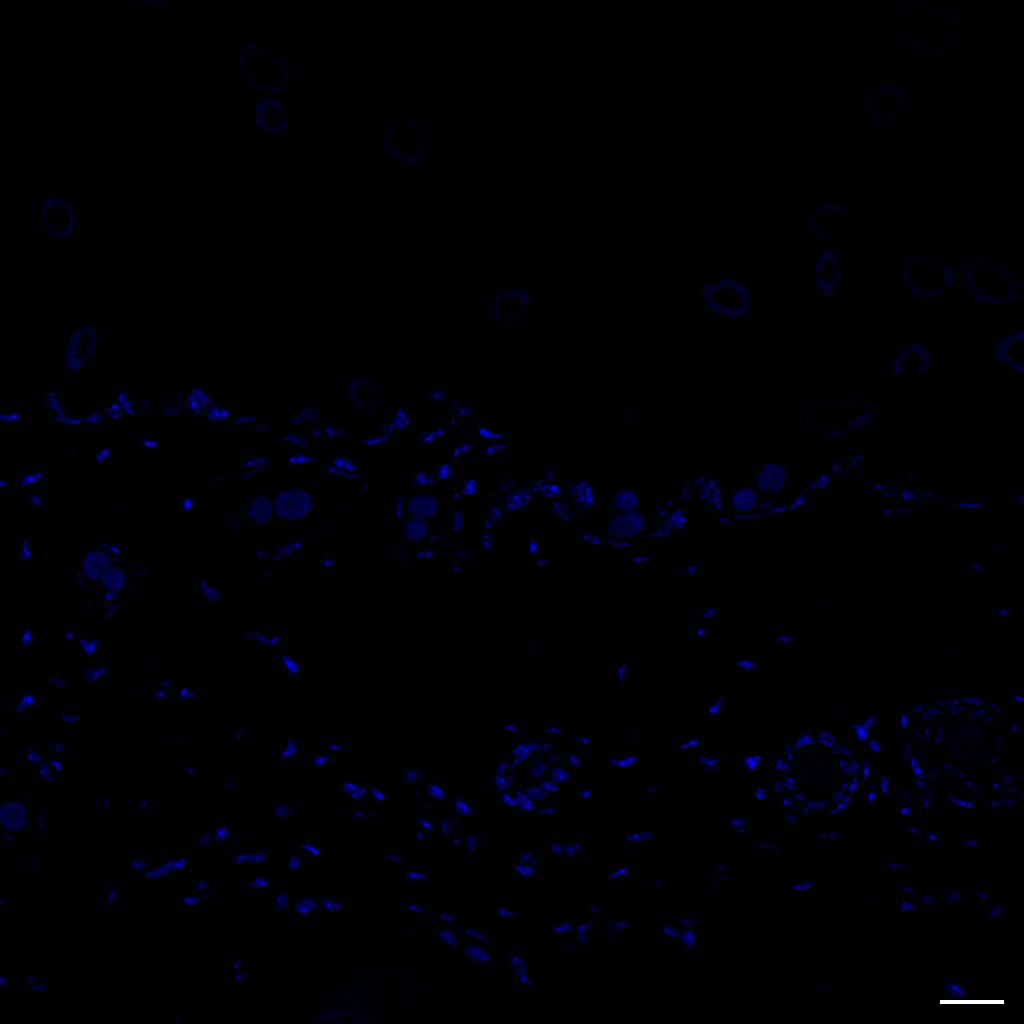

Supplement: Supplementary file 6 — Source Data Fig. 5 [file 44321_2024_48_MOESM6_ESM.zip › Figure 5/5F/EB + daclatasvir skin (r)pSMAD3 dapi.jpg]

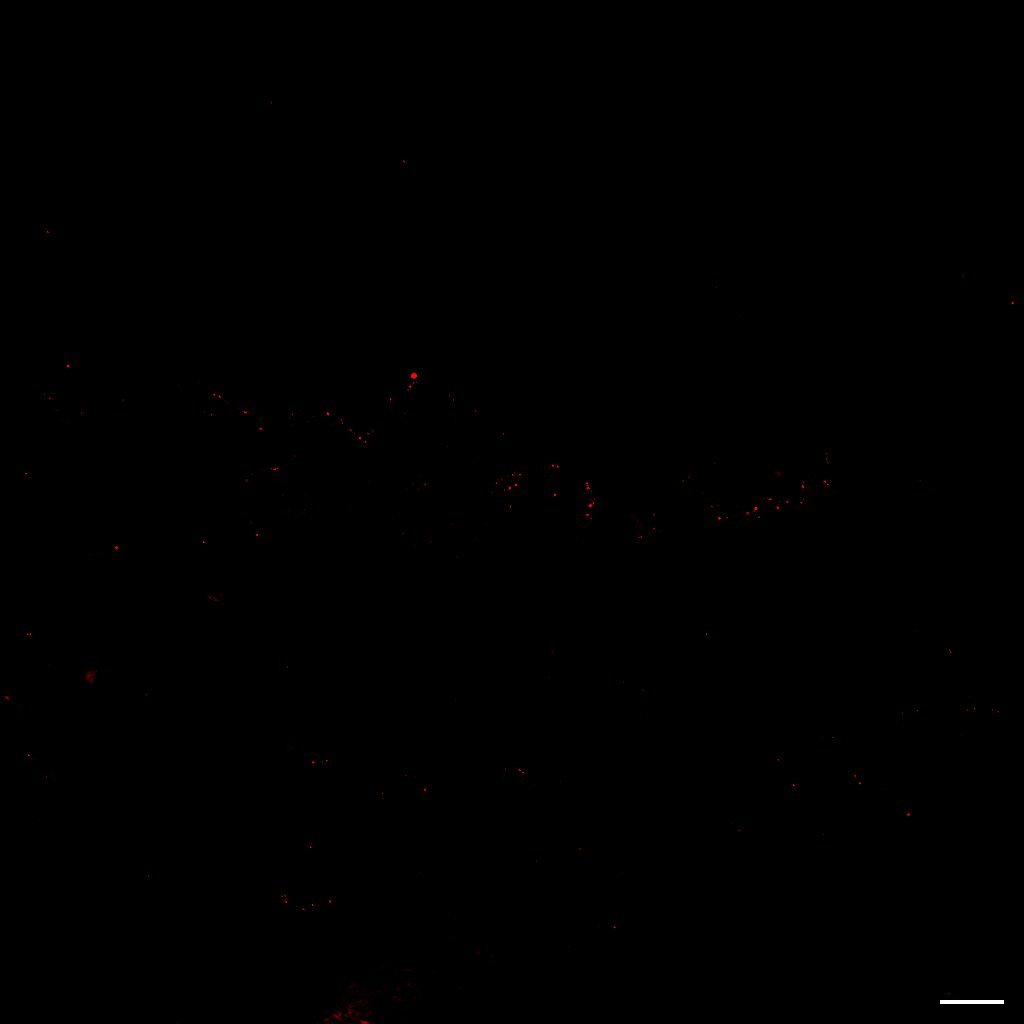

Supplement: Supplementary file 6 — Source Data Fig. 5 [file 44321_2024_48_MOESM6_ESM.zip › Figure 5/5F/EB + daclatasvir skin (r)pSMAD3 only.jpg]

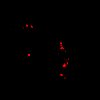

Supplement: Supplementary file 6 — Source Data Fig. 5 [file 44321_2024_48_MOESM6_ESM.zip › Figure 5/5F/EB + daclatasvir skin (r)pSMAD3 zoom in.jpg]

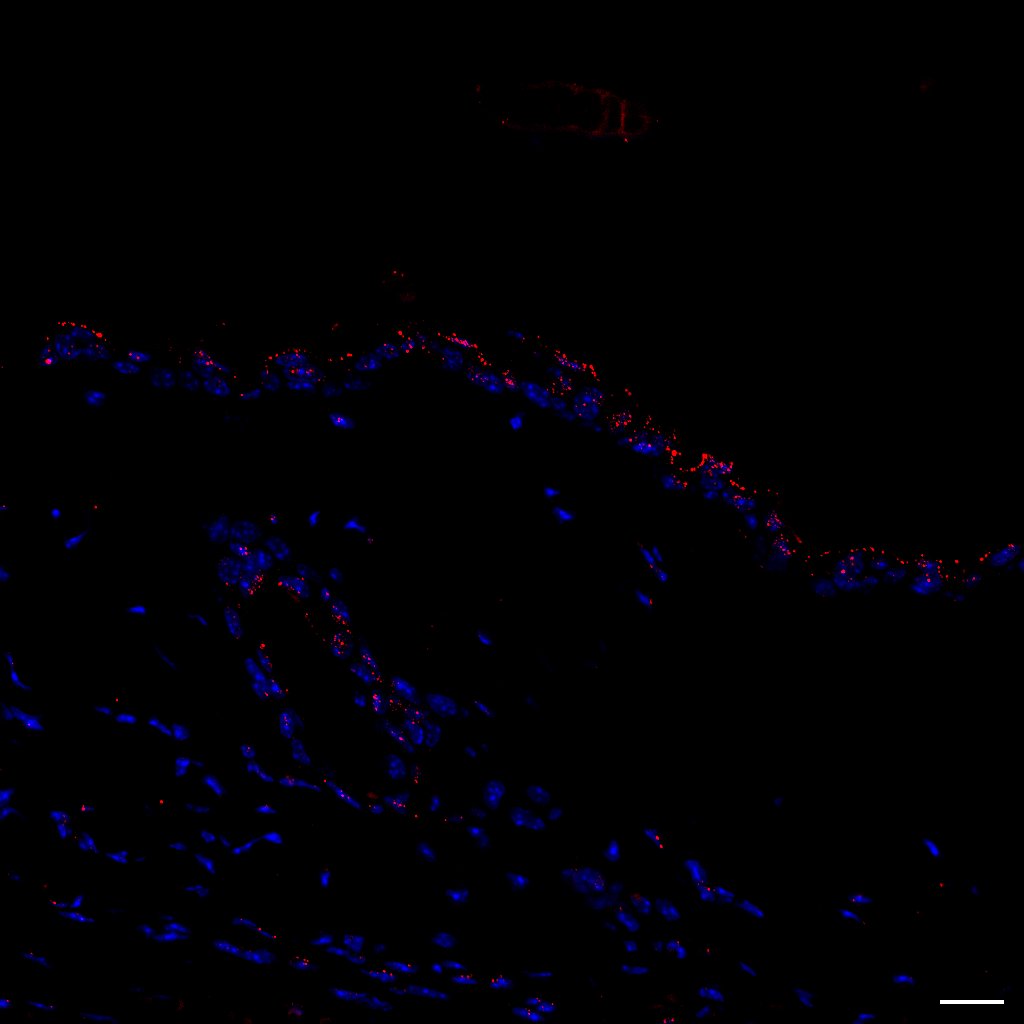

Supplement: Supplementary file 6 — Source Data Fig. 5 [file 44321_2024_48_MOESM6_ESM.zip › Figure 5/5F/untreated EB skin (r)pSMAD3 composite.jpg]

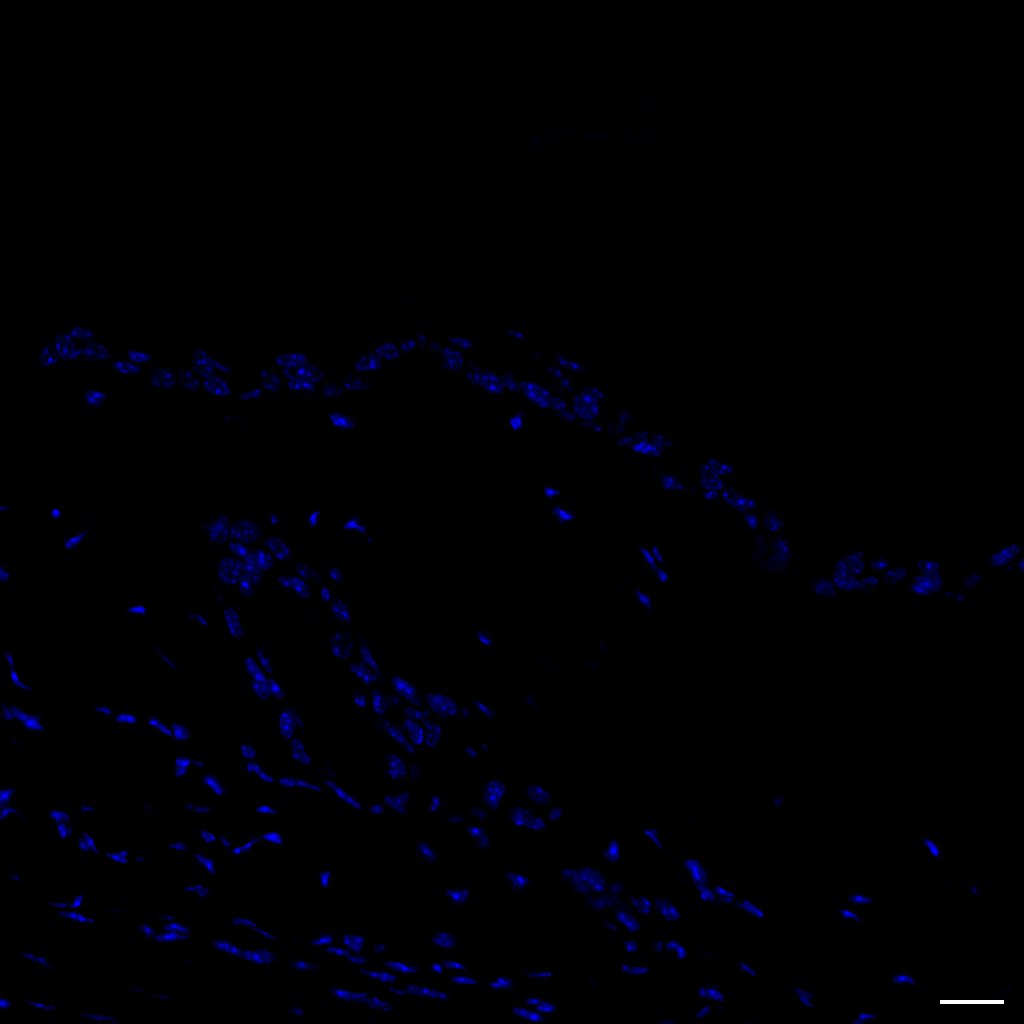

Supplement: Supplementary file 6 — Source Data Fig. 5 [file 44321_2024_48_MOESM6_ESM.zip › Figure 5/5F/untreated EB skin (r)pSMAD3 dapi.jpg]

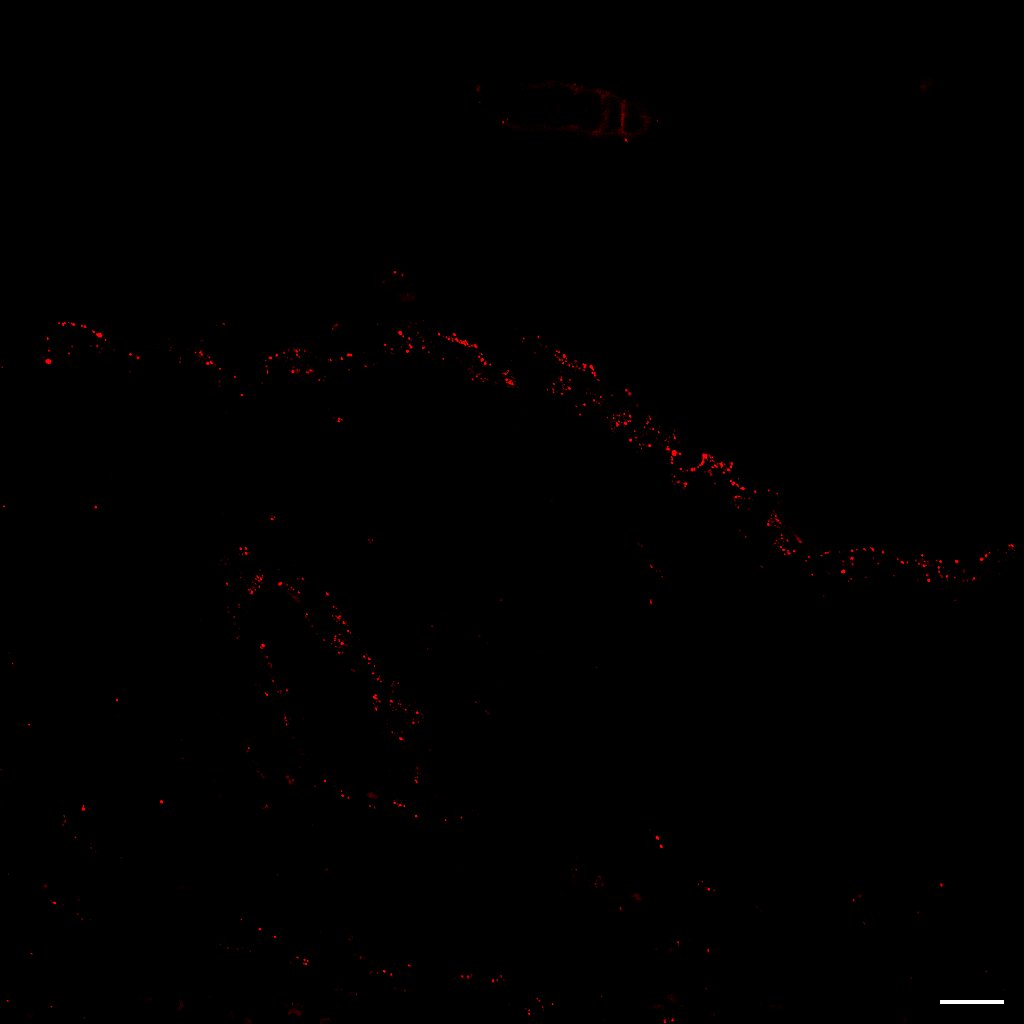

Supplement: Supplementary file 6 — Source Data Fig. 5 [file 44321_2024_48_MOESM6_ESM.zip › Figure 5/5F/untreated EB skin (r)pSMAD3 only.jpg]

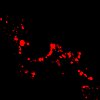

Supplement: Supplementary file 6 — Source Data Fig. 5 [file 44321_2024_48_MOESM6_ESM.zip › Figure 5/5F/untreated EB skin (r)pSMAD3 zoom in.jpg]

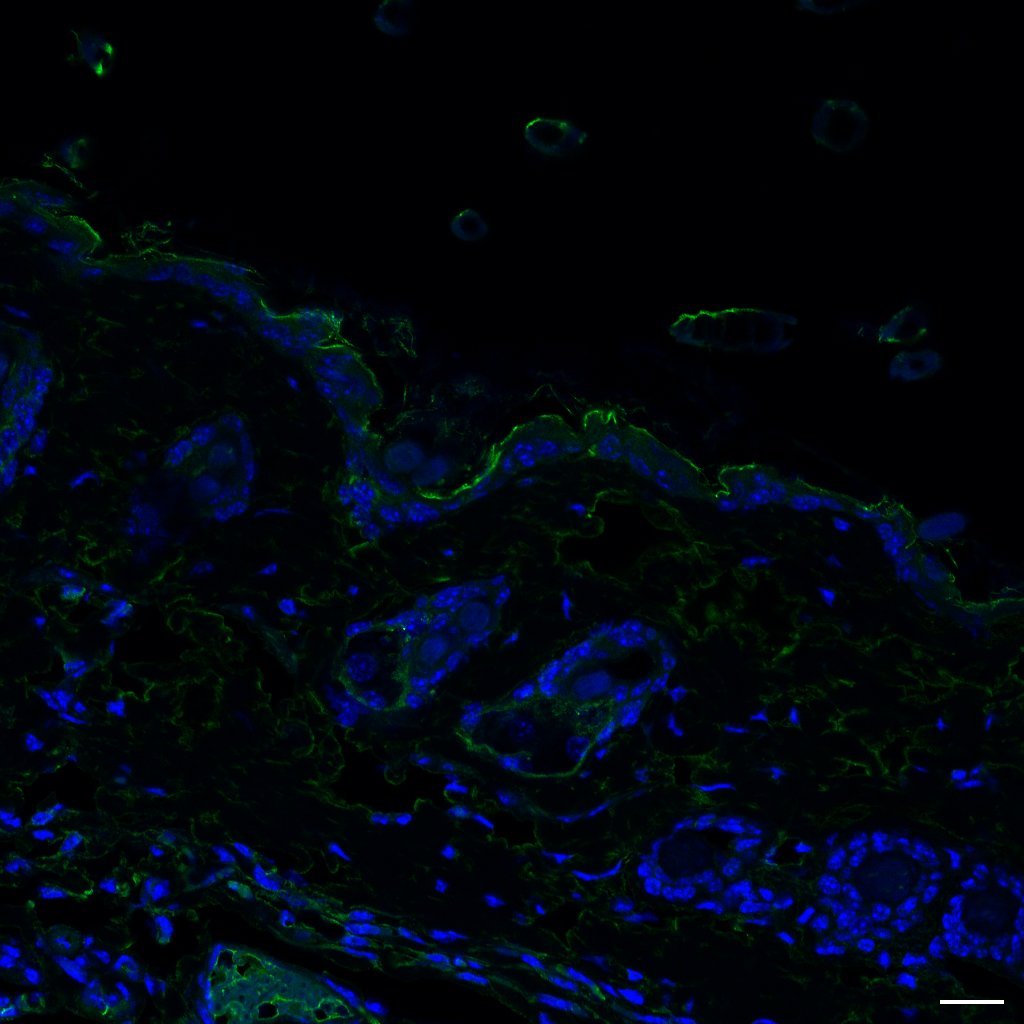

Supplement: Supplementary file 6 — Source Data Fig. 5 [file 44321_2024_48_MOESM6_ESM.zip › Figure 5/5G/EB + daclatasvir skin (g)Collagen I composite.jpg]

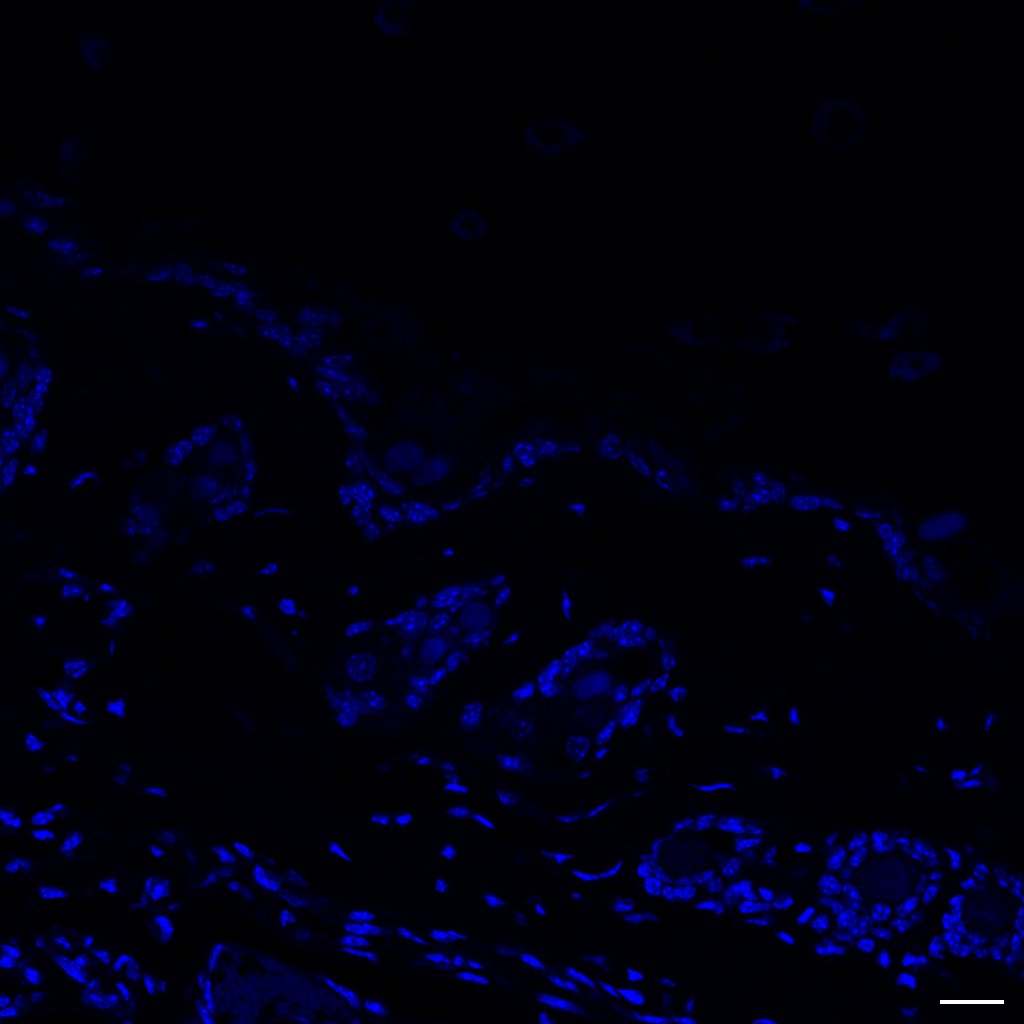

Supplement: Supplementary file 6 — Source Data Fig. 5 [file 44321_2024_48_MOESM6_ESM.zip › Figure 5/5G/EB + daclatasvir skin (g)Collagen I dapi.jpg]

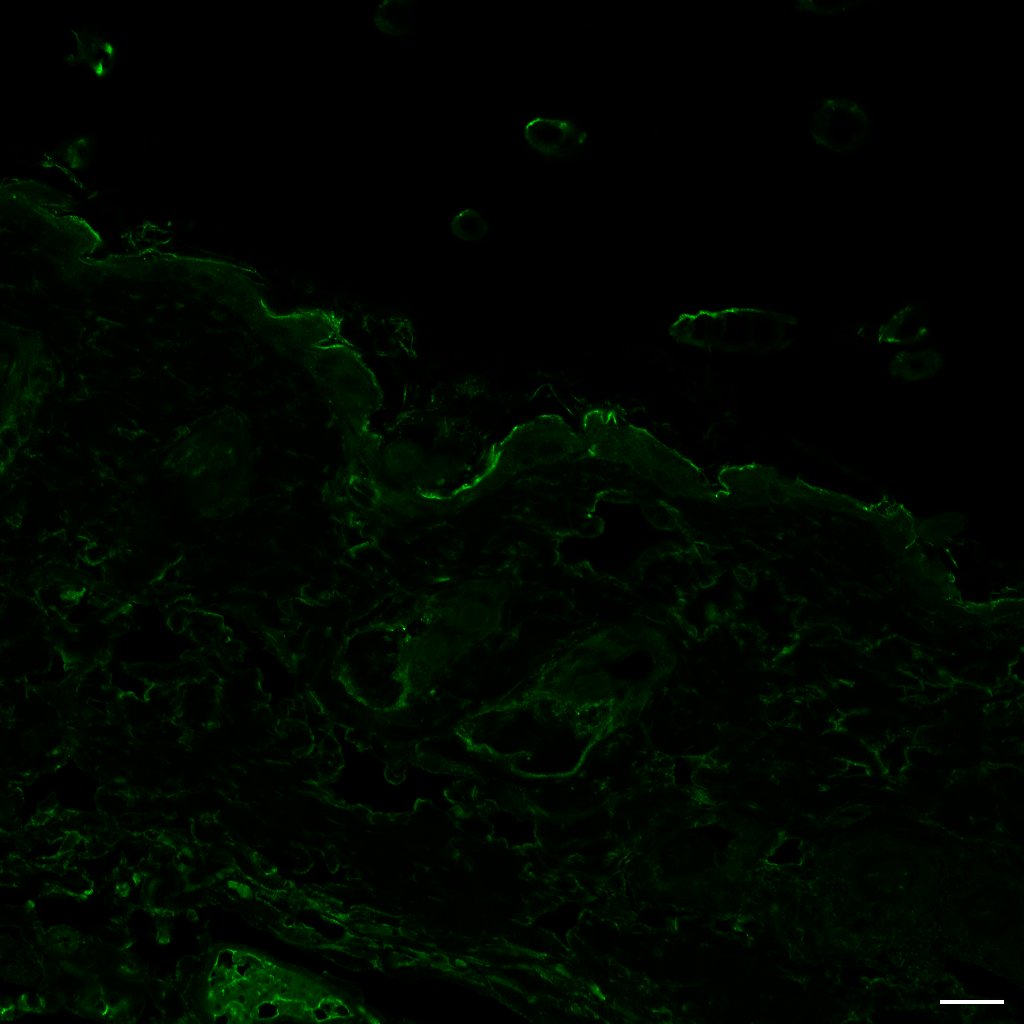

Supplement: Supplementary file 6 — Source Data Fig. 5 [file 44321_2024_48_MOESM6_ESM.zip › Figure 5/5G/EB + daclatasvir skin (g)Collagen I only.jpg]

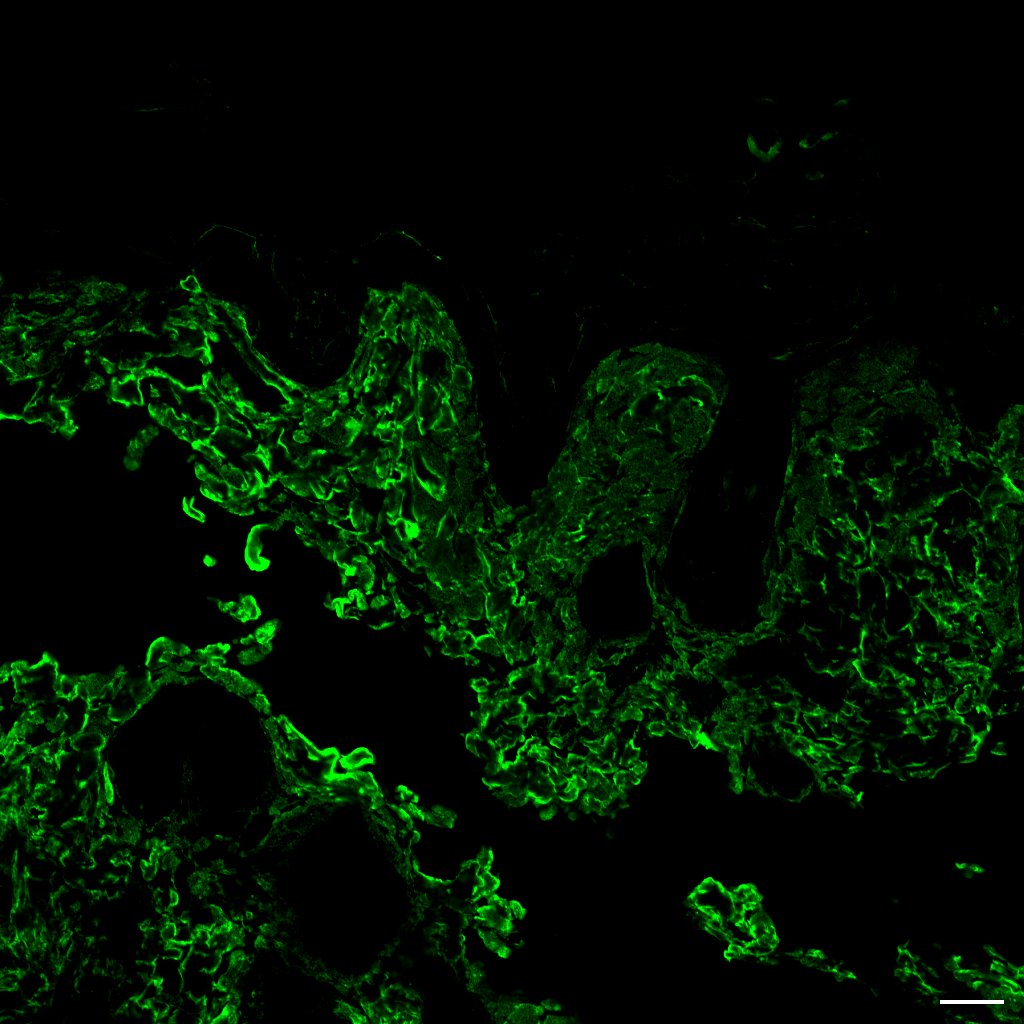

Supplement: Supplementary file 6 — Source Data Fig. 5 [file 44321_2024_48_MOESM6_ESM.zip › Figure 5/5G/untreated EB skin (g)Collagen I only.jpg]

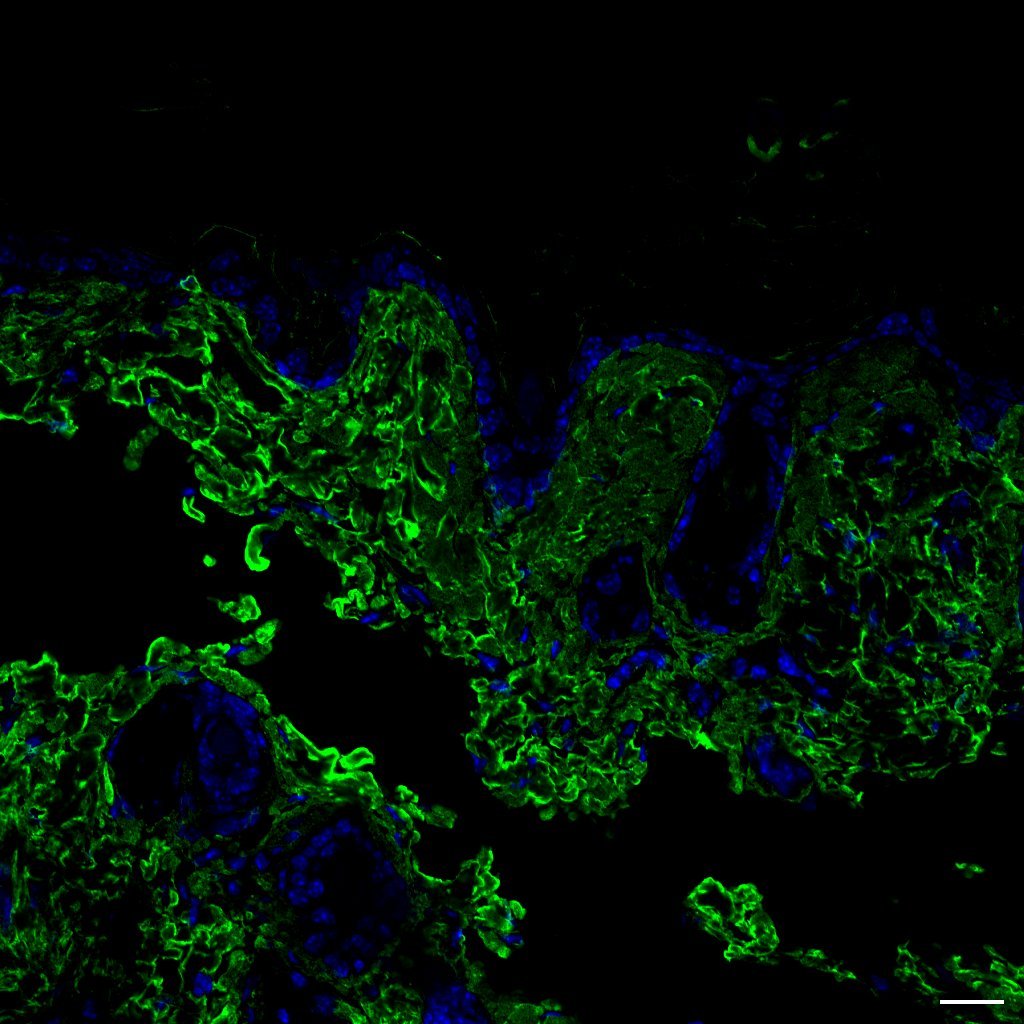

Supplement: Supplementary file 6 — Source Data Fig. 5 [file 44321_2024_48_MOESM6_ESM.zip › Figure 5/5G/untreated EB skin (g)Collagen I composite.jpg]

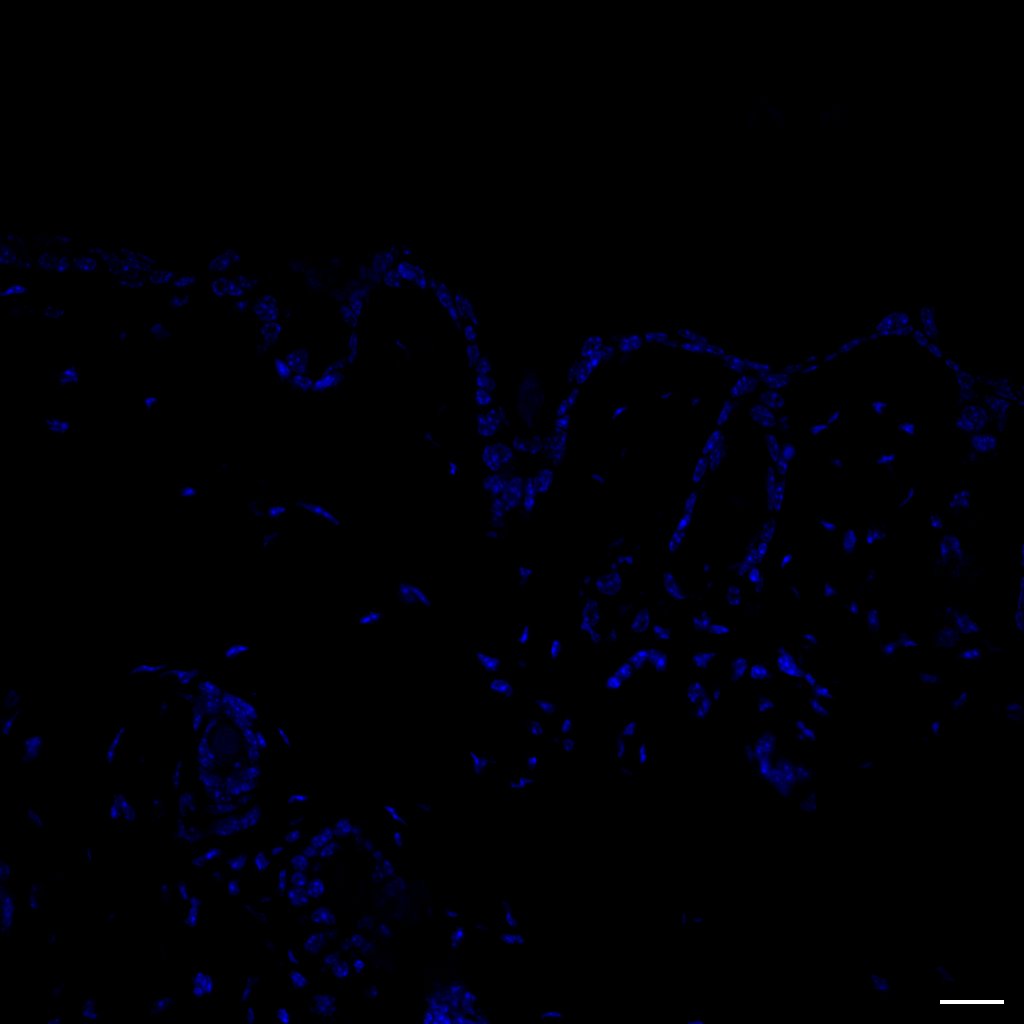

Supplement: Supplementary file 6 — Source Data Fig. 5 [file 44321_2024_48_MOESM6_ESM.zip › Figure 5/5G/untreated EB skin (g)Collagen I dapi.jpg]
